# 3D Printing, Ink Casting and Micromachined Lamination (3D PICLμM): A Makerspace Approach to the Fabrication of Biological Microdevices

**DOI:** 10.3390/mi9020085

**Published:** 2018-02-15

**Authors:** Avra Kundu, Tariq Ausaf, Swaminathan Rajaraman

**Affiliations:** 1 NanoScience Technology Center (NSTC), University of Central Florida, Orlando, FL 32826, USA; avra.kundu@ucf.edu (A.K.); tariq.ausaf@knights.ucf.edu (T.A.); 2 Department of Electrical & Computer Engineering, University of Central Florida, Orlando, FL 32826, USA; 3 Bridging the Innovation Development Gap (BRIDG), Neo City, FL 34744, USA; 4 Department of Material Science & Engineering, University of Central Florida, Orlando, FL 32826, USA

**Keywords:** makerspace microfabrication, microelectrode arrays (MEA), microneedles (MNs), microfluidics (MFs), 3D printing, biological microdevices, ink casting, micromachined lamination

## Abstract

We present a novel benchtop-based microfabrication technology: 3D printing, ink casting, micromachined lamination (3D PICLμM) for rapid prototyping of lab-on-a-chip (LOC) and biological devices. The technology uses cost-effective, makerspace-type microfabrication processes, all of which are ideally suited for low resource settings, and utilizing a combination of these processes, we have demonstrated the following devices: (i) 2D microelectrode array (MEA) targeted at in vitro neural and cardiac electrophysiology, (ii) microneedle array targeted at drug delivery through a transdermal route and (iii) multi-layer microfluidic chip targeted at multiplexed assays for in vitro applications. The 3D printing process has been optimized for printing angle, temperature of the curing process and solvent polishing to address various biofunctional considerations of the three demonstrated devices. We have depicted that the 3D PICLμM process has the capability to fabricate 30 μm sized MEAs (average 1 kHz impedance of 140 kΩ with a double layer capacitance of 3 μF), robust and reliable microneedles having 30 μm radius of curvature and ~40 N mechanical fracture strength and microfluidic devices having 150 μm wide channels and 400 μm fluidic vias capable of fluid mixing and transmitted light microparticle visualization. We believe our 3D PICLμM is ideally suited for applications in areas such as electrophysiology, drug delivery, disease in a dish, organ on a chip, environmental monitoring, agricultural therapeutic delivery and genomic testing.

## 1. Introduction

Microfabrication technologies for nanobiosensors, biomedical micro-electro-mechanical systems (BioMEMS) and micro-total analysis systems (MicroTAS) applications have been transitioning away from traditional cleanroom based techniques for rapid, cost effective fabrication of components that will increase access to end users of these devices: Chemists, medical and life science professionals [1]. Makerspaces provide a growing alternative to clean rooms toward the fabrication of such devices as they provide low-cost access to fabrication equipment such as 3D printers, laser cutters, plotters, micromills, ovens, lamination press and other benchtop equipment thereby attracting a diverse community of artists, engineers, product designers and next generation researchers. Such a makerspace environment is particularly attractive for biomedical devices as they provide easy access in an intimidation free environment to application developers that utilize these devices thus providing immense flexibility in varied materials and allowing for rapid design changes with scalable fabrication, since most biological devices do not require the sophistication of the cleanroom environment. Of all the equipment mentioned above, 3D printers are an absolutely essential piece of equipment for makerspaces as they have the capability of creating micro-scale parts and/or tools through an additive, layer-by-layer manufacturing approach at speeds much faster than traditional manufacturing methods. There are various types of 3D printers based on technologies such as stereolithography (SLA), digital light processing (DLP), fused deposition modeling (FDM), selective laser sintering (SLS), selective laser melting (SLM), electronic beam melting (EBM), laminated object manufacturing (LOM) and 2-photon polymerization (2PP) [2]. As a rapidly growing technology applied to research and commercial settings in micro and nanofabrication, 3D printing has been used to demonstrate devices such as wireless sensors [3], soft electronics [4], light controlled 3D micromotors [5], free standing liquid metal microstructures [6], microfluidics [2,7,8], smart objects with embedded electronic sensors and systems [9] etc. It is important to note here that the 3D printers being used can vary from being highly customized [2,4,7,8], expensive systems [3,5] to portable benchtop inexpensive solutions [9] based on the technology adopted for the printer and the resolution of the final print. Compared to most of the aforementioned reports, we have used an inexpensive, benchtop SLA based system and optimized the print processes with standard resins available in this printer for the work reported in this paper. This clearly distinguishes us from other researchers in this space.

Out of the numerous biological devices, microelectrode arrays (MEAs), microneedles (MNs) and microfluidics (MFs) have found tremendous research and commercial interest in various applications such as disease diagnostics, genomic testing, “diseases in a dish and organ on a chip” models, toxicity screenings, transdermal drug delivery, high throughput assays, therapeutics, and other application areas [10,11,12,13,14,15,16]. These devices fall under the broad category of ‘biological microdevices’ and their fabrication until recently has involved the use of sophisticated cleanroom equipment [14,15,16] or the aforementioned sophisticated, customized and expensive 3D printers [2,3,4,5,7,8].

Microelectrode arrays (MEAs) are advancing both medicine and science. Manipulation of electric activity of the brain and the heart are being enabled by today’s first generation microelectrodes. Today’s MEAs are especially important in an in vitro setting since treatments from animal models rarely translate well to humans. But with the help of today’s MEAs and “on demand” human cells (such as induced pluripotent stem cells, iPSCs), researchers are able to create complex diseases in a dish [17,18]. MEAs are traditionally fabricated utilizing silicon, glass, polymers and printed circuit board (PCB) substrates [19,20] and with disposability becoming a key consideration, introduction of rapid prototyping technologies utilizing makerspace processes for MEA fabrication is the logical next step. To the best of our knowledge, MEAs have not been realized by using makerspace processes as a close synergy between the various equipment in a typical makerspace is required to enable micron scale precision which is essential for such devices. The realization of MEAs would essentially require other processes to build upon the 3D printed substrate appropriate for MEA fabrication. Processes such as realization of conductive traces, lamination/casting, laser micromachining/micromilling although available in some makerspaces would still have to be carefully engineered to fabricate MEAs having similar performance to their commercial counterparts. Thus, addressing this growing need to tailor the advanced processes in a makerspace environment for the realization of cost effective, disposable MEAs with rapid translation from design to a manufactured device would enable technologists to keep pace with applications’ developers.

Microneedles (MNs) are utilized to enhance transdermal delivery of small and large molecules by creating micron sized pores in the skin to advance the delivery of a drug across the barrier [15]. MNs are ideal for patient adherence compared to oral delivery as they are minimally invasive, typically do affect pain nerves and offer the potential for self-administration [15]. Traditionally, MNs are fabricated out of substrate materials such as silicon, glass, metals and more recently by micromolding technologies on a variety of polymers. These techniques have issues ranging from reproducibility (silicon) to non-scalability (glass and metals) to requiring multi-level processing (polymers and metal) [21,22,23,24,25,26,27,28,29,30,31,32,33,34,35,36,37,38,39,40]. One promising method for microneedle fabrication that has developed in recent years is a rapid prototyping process known as 2-photon polymerization [41]. However, to print accurately at a rapid rate a system would need highly customized and expensive add-ons such as galvano-scanner and piezoelectric stages. The galvano-scanner is used to fabricate a single microneedle while the piezoelectric stage is used to translate a surface in two dimensions for the fabrication of microneedle arrays. Although the 2-photon polymerization technique provides much better structural resolution and quality than SLA-based 3D printing, it is much more expensive and not typically present in makerspace environments. Further, the entire build area is restricted to 10 cm × 10 cm [42]. Multi-material microstereolithography (μSL) with digital micromirror device (DMD) based projection system can also be used to realize MNs with radius of curvature of 10 μm but the system is highly customized and uses around ~786,000 micromirrors as a part of the DMD device [43]. MNs having a radius of curvature of 25 μm have additionally been fabricated using a similar digital light processing (DLP) 3D printer on personalized curved surfaces for dual-pronged treatment of trigger finger [44].

SLA-based 3D printing, on the other hand can result in a single step process with standard off the shelf resins that converts engineering design data directly into complex three-dimensional products in a layer-by-layer fashion using photopolymerization of resins and is ideal for the creation of MNs. However, the boundaries of commercial SLA based printing technologies need to be pushed further with proper design, printing conditions and post-processing to realize MNs having low radius of curvature.

The field of point of care (POC) microfluidic diagnostics devices have been predominantly addressed in academia with polydimethylsiloxane (PDMS) devices manufactured using soft lithography [16]. However, the fabrication process typically involves substantial human labor and the layered molding process limits the 3D complexity of the devices that can be produced [2]. 3D printing has recently attracted attention as a way to fabricate microfluidic systems due to its automated, assembly-free 3D fabrication, rapidly decreasing costs, and fast-improving resolution and throughput. Alternative rapid prototyping methods that take advantage of makerspace equipment is on the rise in this field and 3D printing based approaches are demonstrating tremendous promise for microfluidic device fabrication [45]. While these methods do not provide superior resolution of photolithographic methods for now, the use of plastic, paper, and laminate substrates provides a pathway for cost effective, large area manufacture of POC based diagnostic microfluidic devices. It is important to note here that the resolution offered by standard, commercial non-customized, SLA-based 3D printers is limited by the laser spot size (typically around 100–150 μm) and the absorption spectra of the photoresins [2]. However, while printing microfluidic channels this resolution is highly compromised resulting in dimensions in the order of a few millimeters [46]. This is simply because that the walls of the fluidic channel are fused with the rest of the model during the print process. Highly customized [2,4,7,8], expensive systems [3,5] as demonstrated by some researchers can typically lower the print resolution but novel design schemes when coupled with other makerspace processes can also allow for improved resolution of microfluidic channels printed with benchtop SLA printers.

In this paper, we present a complete makerspace, benchtop microfabrication technology and utilize this method to develop and characterize three distinct biological microdevices: MEAs, MNs and multi-layer microfluidics by using materials and equipment that present the promise for low cost, high accessibility, simplicity, scalability, and large area manufacturing. The technology involves the use of an inexpensive, benchtop SLA 3D printer, selective ink casting and micromachined (μM) lamination processes and is entitled 3D PICLμM. Selective ink casting defines conductive traces and lamination of biocompatible adhesives/films further defines an insulation layer. Both of these layers are built upon an additive manufacturing base provided by a 3D printer. Further, our process involves an intimate symbiosis between additive manufacturing and subtractive technologies that enable micron-scale precision (something that has evaded non-customized, benchtop additive technologies thus far) [47]. As a result, our 3D PICLμM process leverages the rapid, cost effective fabrication advantages of additive technologies to produce arbitrary non-planar shapes and curved faces that would be difficult to obtain utilizing traditional micromachining technologies. The additive steps are followed by the use of the advanced precision of subtractive technologies to remove material as needed to produce a myriad of biological microdevices. Such a combined process technology has the power to diversify and consolidate the varied application fields for printed microscale devices thus realizing MicroTAS and BioMEMS devices in bio-functional polymers and resins.

## 2. Materials and Methods 

The process of 3D PICLμM uses various makerspace steps in different combinations to fabricate the biological microdevices. These steps include 3D printing, with the ink casting, lamination and micromachining processes adding various functionalities to the 3D printed device. 3D PICLμM is hierarchical in nature with each unit process building upon the functionalities provided by the earlier process. The ink casting process can be used to define conductive tracks selectively on the 3D printed devices and the lamination process may either act as packaging or insulating layers added to the 3D printed base and/or the selectively defined conductive tracks. The micromachining techniques allow for subtractive processes in 3D PICLμM enabling us to obtain a synergy with the additive manufacturing steps. Subtractive definition of features is obtained with a micro-drill bit or laser micromachining based on the requirement of the final device. Out of the three distinct devices, the fabrication of the microneedles requires only 3D printing while the microfluidic channel realization requires 3D printing and lamination processes and the MEAs involve 3D printing, ink casting, lamination and micromachining processes. All the devices were designed in Solidworks (2016 x64 bit edition, Dassault Systems Inc., Waltham, MA, USA) which allows us to generate design concepts rapidly and offers unique tools for the creation, manipulation, and modification of designs using native and imported geometries. The designed stereolithography (SLA) file is printed in Form Labs Form 2 (Somerville, MA, USA; SLA 3D printer with a laser wavelength of 405 nm) using a photopolymer clear resin (FLGPCL02, Formlabs, Somerville, MA, USA). The X- and Y-resolution of the laser is determined by the spot size of the laser which is 140 μm. The axial resolution in Z direction was kept at 25 μm. The processes and materials used to create the biological microdevices are described below for the individual devices.

### 2.1. Microelectrode Arrays

#### 2.1.1. Design and 3D Printing

**Figure 1:**
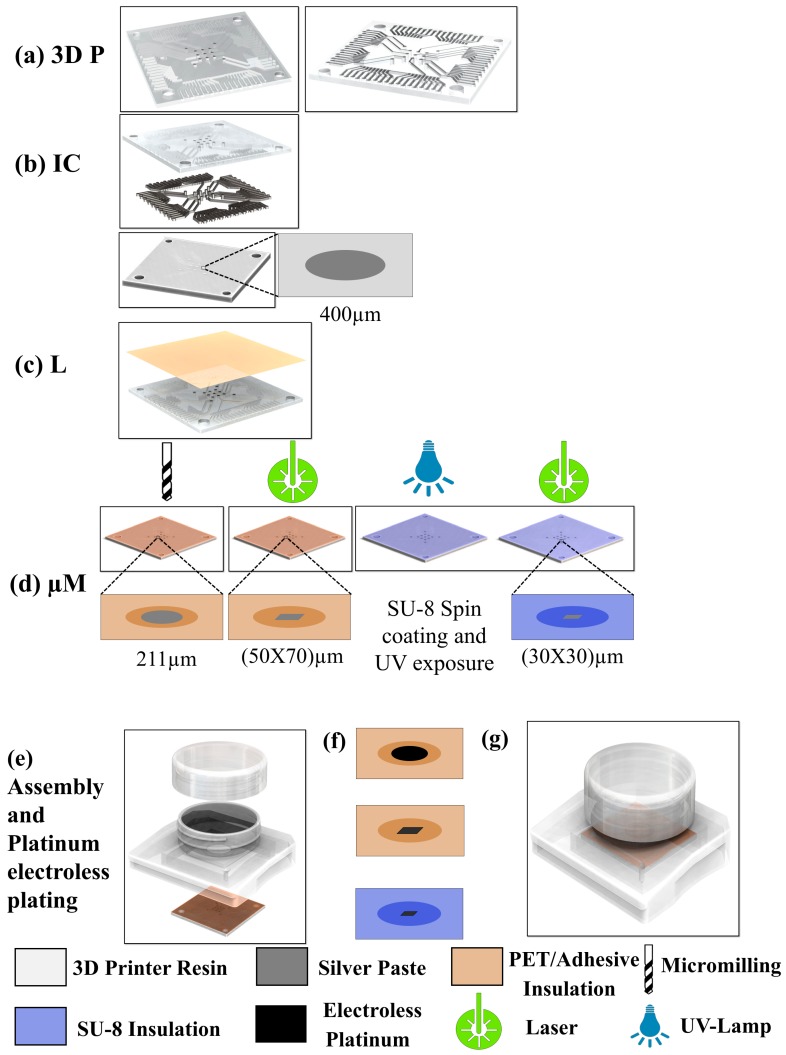
(**a**) 3D design of the microelectrode array (MEA) used for 3D printing (3D P); (**b**) ink casting (IC) of silver paste on the bottom face of the MEA; (**c**) lamination (L) of an insulation layer or casting/curing of SU8; (**d**) micromachining (μM) process by micromilling or laser micromachining; (**e**) assembly and platinum electroless plating for the definition of low impedance electrodes; (**f**) individual electrodes of different sizes after platinum electroless plating; (**g**) final, fully assembled device ready for testing.

Figure 1a shows the 3D design of the MEA targeted in this work. It has a unique non-planar design with a monolithic construction of the electrode tracks, electrode landing pads and vias both on the top and bottom faces of the MEAs. Such a non-planar design allows us to isolate the electrode tracks from the top side of the MEA thereby improving device reliability due to shorting or damages to the insulation layer. The design has a total of 9 working microelectrodes and 4 integrated reference ground electrodes and is 1 mm thick. The electrode vias are 400 μm in diameter and the pitch between the electrodes is 1 mm. This particular design of the microelectrodes is targeted at applications in precision plating of cells [48]. The width of the conducting traces is 200 μm with a depth of 100 μm. The electrode conducting traces terminate into contact pads which have a width of 350 μm, length of 1 mm and pitch of 350 μm. The contact pads are designed to interface with the Axion BioSystems (Atlanta, GA, USA) commercial MUSE electronics (Axion BioSystems, Atlanta, GA, USA) and AxIS software (version 2.3, Axion BioSystems, Atlanta, GA, USA). To obtain an optimized print quality of the various features, the MEA design was printed at 30°, 45°, 60° and 90° with respect to the anchor/substrate holder (described in Section 3.1.1). The printed devices were rinsed twice in isopropyl alcohol (Sigma-Aldrich, St. Louis, MO, USA) for 10 min and were dried with a nitrogen gun. The devices were not temperature cured in order to avoid deformation of the devices.

#### 2.1.2. Ink Casting

Ink casting (Figure 1b) was performed to define conductive traces using Epo-tek^®^ EJ2189 (Epoxy Technologies Inc., Billerica, MA, USA), an electrically conductive (resistivity values range: 0.0005–0.009 Ohm-cm), silver filled epoxy paste suitable for low temperature curing from ambient to 80 °C. The conductive ink was coated with a cotton swab (Pur-Wraps^®^, Puritan Medical Products, Guilford, ME, USA) onto the entire bottom face of the 3D printed device with the MEA geometry. The paste was subsequently removed utilizing a different cotton swab from the device area which leaves the paste in the micro-troughs (residing at 100 μm below the surface) intended for the conductive traces and conductive vias due to the difference in height between the top and bottom levels of the paste. The devices were cured at 40 °C for 3 h, subsequently rinsed in isopropyl alcohol and dried with a nitrogen gun. At this stage, the device may be used as a MEA with microelectrodes that have a diameter of 400 μm due to its unique non-planar design.

#### 2.1.3. Lamination

To fabricate smaller microelectrodes, the lamination (Figure 1c) of an insulation layer or casting/curing of SU-8 is performed on the devices and electrode openings are obtained by using subtractive processes such as micro-drilling or laser micromachining (Figure 1d). For laminating an insulating layer (Medco^®^RTS3851-17 adhesives ~50 μm thick plus poly ethylene terephthalate (PET) ~20 μm thick; Medco Coated Products, Cleveland, OH, USA) was utilized. PET is biocompatible and has been used successfully as a substrate for MEAs previously by the authors [49]. The liner on the adhesive layer was removed and it was affixed to the device. The lamination layer was defined to its final shape utilizing a pair of scissors. The ink cast 3D printed device and the PET/adhesive layer were pressed with 100 pounds of force at room temperature for 30 s in a manually operated, benchtop hydraulic laminating press (Carver, Inc., Wabash, IN, USA). The final thickness of the insulation layer including the adhesive and the PET layer is approximately 63 μm after the pressing step. This lamination technique is remarkably fast and simple.

For defining thinner insulation layers comparable with commercial MEA devices [50,51], SU-8-negative tone photo-epoxy (GM 1050 from Gersteltec, Pully, Switzerland) was used. The devices were spin coated at 1660 rpm for 40 s with a ramp of 100 rpm after application of the photoresist to achieve a uniform thickness of approximately 5 μm. The samples were soft baked at 40 °C for 10 min, followed by the UV flood exposure (365 nm) of the samples to completely crosslink the SU-8 photoresist using a UVP Blak-Ray™ B-100A (Upland, CA, USA) UV lamp for 3 min. A post exposure bake was performed at 40 °C for 5 min.

#### 2.1.4. Subtractive Processes for Definition of MEA Recording Sites

Micromilling and laser micromachining were chosen as the fabrication processes for the definition of recording sites on the insulation layer. Figure 1d elaborates these micromachining processes.

(a) Micromilling: For the fabrication of the microelectrode recording sites, an approximately 211 μm thick drill bit (T-Tech, Peachtree Corners, GA, USA) was spun at 55,000 rpm in a T-Tech J5 Quick Circuit Prototyping Systems. The total drilling time was 39 s considering a drilling speed of 180 holes/min and 13 drilling sites in a single MEA. This lamination/micromilling technique for definition of the recording sites is significantly shorter than a standard lithographic technique.
(b) Laser Micromachining: For the fabrication of the microelectrode recording sites, defined on the laminated adhesive using the laser, a green laser (532 nm) with a spot size of (70 μm × 50 μm) was fired at an energy level of 50 mJ at a repetition rate of 50 Hz using a QuickLaze 50ST2 (Eolite Lasers, Portland, OR, USA). The laser spot was aligned atop the lamination areas on the microelectrodes prior to the deployment of the laser. For the definition of the recording sites in the SU-8 layer, the green laser was fired with a spot size of (30 μm × 30 μm) with 25 mJ energy at a repetition rate of 50 Hz.

#### 2.1.5. Packaging

For packaging of the devices, culture wells with caps were designed utilizing Solidworks and 3D printed utilizing the Form Labs Form 2 printer. For attaching the fabricated MEAs with the culture well, Epo-tek^®^ 353ND was used. Parts A and B of the epoxy were mixed in ratio of 10:1 (by weight) and applied to the underside of the culture well and the fabricated MEAs were assembled face down. The packaged device was cured at 40 °C for 4 h. The devices were tested for any leaks with ethanol and DI water prior to the electrical and electrochemical measurements. Figure 1e depicts a schematic of the various components for assembling the final device.

#### 2.1.6. Electroless Plating

For electroless deposition of the microelectrode material (porous platinum), 0.01 weight percentage (wt %) platinum solution was prepared using 3.75 mL (~8% chloroplatinic acid from Sigma-Aldrich), 0.2 mL of 0.005 wt % lead acetate (Sigma-Aldrich), 4.065 mL of 1.23 M HCl (Sigma-Aldrich) and 2.085 mL of DI water. Approximately 3 mL of this solution was transferred to the MEA culture well and passive electroless plating was performed for 1, 3 and 6 h respectively to estimate the time required for obtaining complete platinum coverage on the electrodes. Optical observations of the electrolessly plated platinum were performed at the pre-defined time periods utilizing a microscope after the MEA device was rinsed with DI water and the liquid was removed with nitrogen blow drying. Figure 1f depicts a schematic of the individual electrodes of different sizes after the electroless plating of porous platinum.

#### 2.1.7. Electrical and Electrochemical Measurements

Impedance measurements of the MEAs were performed with the final device (Figure 1g) using Bode 100 (Omicron Labs, Houston, TX, USA) with Dulbecco’s Phosphate Buffer Solution (Thermo Fisher Scientific, Waltham, MA, USA) as the electrolyte. The impedance scans were carried out from 10 Hz to 1 MHz with a platinum wire (eDAQ, Denistone East, Australia) as the counter electrode. Cyclic voltammetry (CV) was performed using Potentiostat 466 system (from eDAQ). The CV measurements are performed with a 3-electrode setup with a silver/silver chloride (Ag/AgCl) wire acting as the reference electrode and a Pt wire used as the counter electrode with Dulbecco’s phosphate buffered saline (PBS) (1×) as the electrolyte. CV scans were performed from −1V to 1V with scan rates of 50 mV/s, 100 mV/s, 160 mV/s, 200 mV/s and 250 mV/s to estimate the capacitance of the electrodes.

#### 2.1.8. Imaging

Optical imaging of the microelectrodes was performed in BX51M microscope (Olympus, Center Valley, PA, USA). Scanning electron microscope (SEM) imaging and EDS analysis of the printed devices and the electrolessly deposited Pt. were performed using JSM 6480 (JEOL, Peabody, MA, USA).

### 2.2. Microneedles

#### 2.2.1. Design and 3D Printing

**Figure 2:**
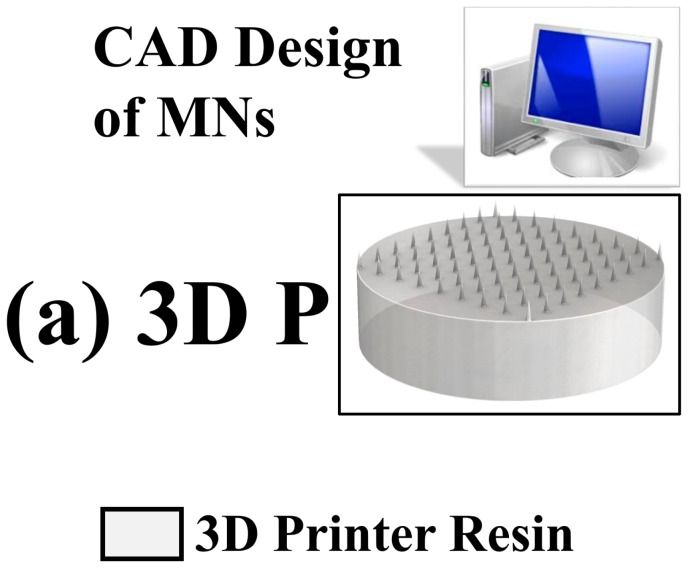
3D design and fabrication of the microneedles targeted in this work involving only CAD design and 3D printing (3D P).

Figure 2 shows the schematic of the solid microneedles (MNs) targeted in this work. The microneedle patch has a diameter of 25 mm and a thickness of 5 mm with an array of 81 microneedles arranged in a 9 × 9 matrix. Microneedles of varying aspect ratios (ratio of needle height to needle diameter): 3.33, 3.75 and 4 which correspond to needle height/needle diameter of 500 μm/150 μm, 750 μm/200 μm and 1000 μm/250 μm respectively were designed and printed. To evaluate the accuracy of the angular optimization, a test MN geometry (1000 μm base diameter, 1000 μm height) was printed at angles 0°, 45°, 60° and 90° with respect to the anchor/substrate holder. All MNs were subsequently cured at a temperature of 60 °C for 60 min in an oven to obtain a high tensile strength (65 MPa) [46].

#### 2.2.2. Acetone Vapor Polishing

The fabricated MNs were placed on top of an aluminum foil that was placed inside a 1-liter glass beaker. Kimwipes (Kimtech, Roswell, GA, USA) were soaked in acetone and hung from the interior edges of the beaker. The beaker was sealed with Parafilm^®^, (Sigma-Aldrich) and the microneedles were polished in acetone vapor for 4, 6 and 10 min to obtain optimized polishing times.

#### 2.2.3. Measurements

As the MN array is mainly intended for transdermal drug delivery by self-administration it is essential that the MN array punctures the top layer of skin, the stratum corneum [15]. The puncturing force of the MN array has to be carefully tailored since a smaller force would lead to unsuccessful skin penetration while larger forces could result in pain. The MN array optimization (tip diameter and number of tips) has therefore been carried out in order to optimize a force that can make successful skin penetration with minimal discomfort to an individual. To estimate this optimum value, measurements of applied force were performed using a circular Force-Sensitive Resistor (FSR, Adafruit, New York City, NY, USA) with the help of volunteers. The volunteers were requested to press upon the FSR with the following qualitative metrics: mild, gentle, hard and very hard. The resistance value of the FSR was recorded with the help of a multi-meter and converted to corresponding values of the force applied with the help of calibration graphs provided by the manufacturer [52]. Digital force gauge (Zhiqu Precision Instruments, Dongguan, China DS2 series; 10 N and 50 N) was used to calibrate the FSR. For the calibration experiment, the FSR/MNs was pressed with known values of force from the digital force gauge and the corresponding resistance value was recorded and converted to a force value as explained above.

Microneedle puncture tests were performed on a Human Skin Suture Training Model (Anatomicals IV Terapy Products, Lake Forest, IL, USA) for measurements of microneedle penetration depth and calculation of the optimum force required for uniform microneedle penetration. The applied force was measured using a calibrated FSR below the thumb of an individual pressing the microneedle array onto the Human Skin Suture Training Model. Staining of the epidermis layer was performed using 1% wt. Rhodamine 6G (Sigma Aldrich, St. Louis, MO, USA) in water.

The digital force gauge was additionally used to obtain microneedle fracture data. For the fracture data experiments, the Human Skin Suture Training Model was placed on one of the platens of the digital force gauge and the MN array was affixed with double sided adhesive tape on the other platen. The platens were pressed together at known values of force to fracture MNs and record the value. The MN fracture was observed optically with a microscope and confirmed with SEM measurements. The same tool (SEM) was utilized to quantify the variation of the microneedle tip diameter of a given array during the fracture tests.

### 2.3. Microfluidics (MFs)

#### 2.3.1. Design and 3D Printing

**Figure 3:**
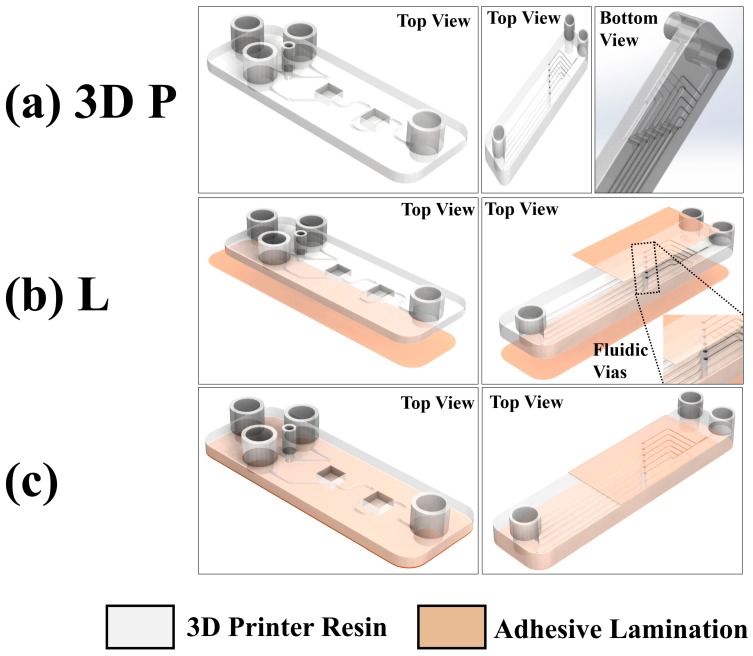
(**a**) 3D design of the microfluidics (MFs) used for 3D printing (3D P); (**b**) lamination (L) with poly ethylene terephthalate (PET)/adhesive to seal the microfluidic channel; (**c**) final fabricated device ready for testing. The top view schematics of the single layer Y-channel device is depicted on the left and the multi-layer device is depicted on the right.

Figure 3 shows the Y-channel microfluidic design realized using the 3D printing and lamination processes. The microfluidic channel is printed as an open channel on the bottom of the device which is subsequently sealed with a transparent adhesive lamination process to define a closed microfluidic channel. Such an approach results in achieving channel dimensions down to 150 μm in width. The two entry and one exit ports allow for dispensing and collecting the fluids respectively. Two view-ports are additionally 3D printed to observe the microchannel, one for viewing the Y-junction (View-Port 1) and the second for monitoring the micromixing process away from the Y-junction (View-Port 2). Further, the process can be extended for double-sided devices with features on both sides of a 3D printed base. Such multi-layer processing can result in sealed channels on both sides of a single substrate interconnected with 3D printed microfluidic vias of 400 μm width. Figure 3a shows the 3D design of the Y-channel microfluidic device and double-sided microfluidic device.

#### 2.3.2. Lamination

The lamination of the MFs were performed in a process similar to that for the MEAs with the PET/adhesive, Medco^®^RTS3851-17. Figure 3b shows the lamination process for the Y-channel microfluidic device and double-sided microfluidic device. The fluidic vias are also shown as an inset in the figure.

#### 2.3.3. Measurements

Gentian Violet (Humco Austin, TX, USA) and 1% wt. Rhodamine 6G (Sigma-Aldrich) were used as color markers to demonstrate fluid flow and mixing in the in the finished microfluidic devices (Figure 3c). Gentian violet and Rhodamine 6G were dispensed into the two entry ports using a graduated pipette. Different concentrations of the PBS buffer solution were prepared by diluting the 1× concentration with Ethanol (Sigma-Aldrich) to obtain dilutions of 0.75×, 0.5×, 0.25× and 0.1× and utilized in the fluidic impedance measurements. Polystyrene (PS) latex beads (1.1 μm diameter, Sigma-Aldrich) were used as a cell-like material for the optical analysis of the microfluidic mixer and the control (PS petri dish) with different concentrations in DI water (1×, 0.5×, 0.25× and 0.125×) was prepared for the imaging experiments.

#### 2.3.4. Imaging

The Y-channel junction was observed through View-Port 1 in the device. Images of the Y-channel depicting micromixing were obtained using a transmitted light microscope (Olympus CK2) for qualitative analysis of the microfluidic design. For estimating the number of latex beads in the microfluidic channels, images were obtained in a dark field mode utilizing View-Port 2 of the device. A similar imaging technique was followed for estimating the number of beads in the control device (PS petri dish).

## 3. Results and Discussions

In this section we detail the results of printing optimization as well as results from the individual devices: MEAs, microneedles and microfluidic devices.

### 3.1. 3D Print Optimization

#### 3.1.1. Effect of Print Angle

**Figure 4:**
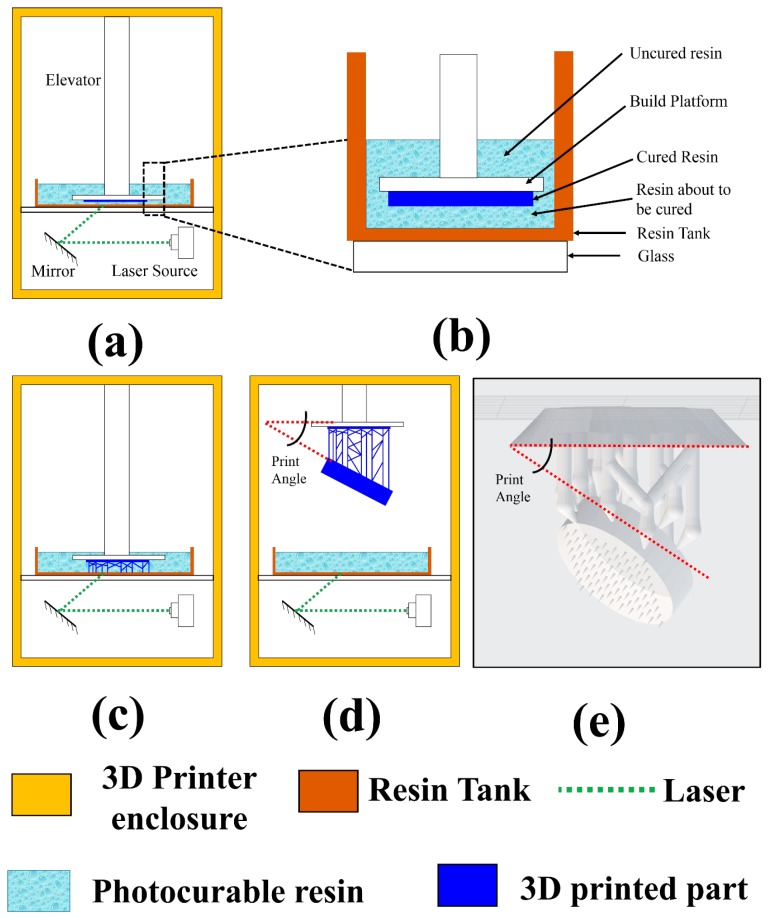
Schematic of our benchtop based 3D stereolithography (SLA) printing with its key components and various printing stages—(**a**) Stage 1: Printing of the base support structure; (**b**) expanded view of the various interfaces in a typical benchtop SLA 3D printer; (**c**) stage 2: Printing of the scaffolds for supporting the actual 3D printed geometry; (**d**) stage 3: Final 3D printed device released and supported by the scaffolds at an angle with respect to the horizontal (print angle); (**e**) Schematic of a typical microneedle prior to printing (the print angle is clearly depicted in the figure).

Figure 4 shows a schematic of the benchtop based 3D SLA printer used in our 3D PICLμM process. In SLA based printing, the 3D object is built layer by layer by using selective light exposure to photo-polymerize a precursor resin collected in a tank. The layer-by-layer printing can be broadly divided into three stages. Stage one corresponds to the printing of the base support structure (Figure 4a). Figure 4b shows an expanded view of various interfaces present in the benchtop based SLA printing. It is seen that the laser must travel through the glass and the resin tank prior to the photo polymerizing the resin. This results in the bending (refraction) and distortion (diffraction) of the laser and limits the resolution of the 3D printing [53,54]. Stage one is followed by printing of the scaffolds for supporting the actual 3D printed device (Stage two shown in Figure 4c). Stage three corresponds to the printing of the actual device geometry supported by scaffolds (Figure 4d). The actual 3D printed geometry can be inclined at an angle with respect to the horizontal for optimized print quality. This angle is quite critical for pushing the limits of standard benchtop SLA printers without the requirement of extensive customization and is indicated in Figure 4d. Figure 4e shows a typical microneedle design prior to printing with the print angle clearly marked in the figure. Such sequential printing results in the building of the object in an upside-down orientation, and is therefore commonly referred to as the “bat” configuration [2].

**Figure 5:**
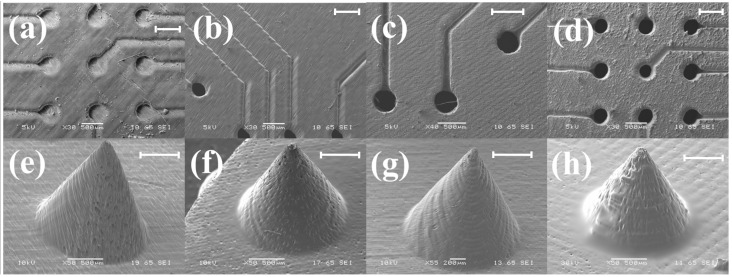
Effect of the angle at which the MEAs and MNs are 3D printed with respect to the horizontal axis. (**a**–**d**) MEAs printed at angles 0°, 45°, 60° and 90° respectively and (**e**–**h**) MNs printed at angles 0°, 45°, 60° and 90° respectively. The scale bars in the figure correspond to a measurement of 500 μm.

Figure 5 shows the effect of the angle at which the MEAs are printed with respect to the horizontal axis. At an angle of 90° with the horizontal, the printed device is geometrically perpendicular to the direction of the laser light. Further, although the axial resolution in the *z*-axis is 25 μm, the laser spot size of the SLA printer is 140 μm (full width half maximum) which makes the laser spot size comparable to the 3D print dimensions for our devices. Thus, as the laser beam is focused onto the surface of the liquid photopolymer to print each layer of the device, the diffraction of the laser beam limits the print resolution resulting in the incomplete definition of the electrical vias and the micro-troughs intended for ink casting of the conducting traces (Figure 5a).

**Figure 6:**
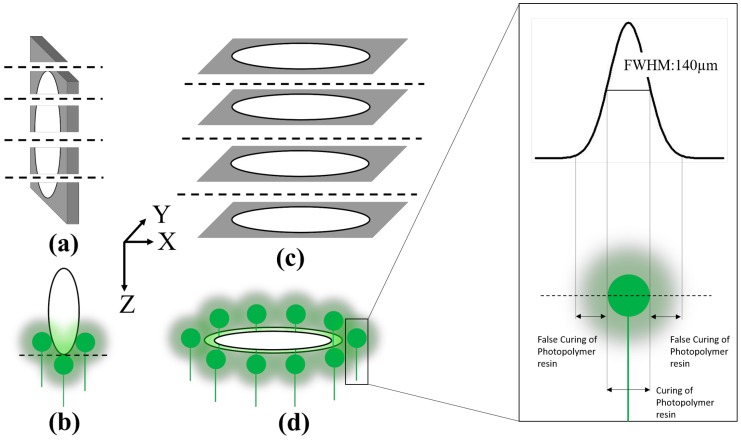
Schematic of our proposed theory of 3D printing—(**a**) Cross-section of only an arc of the electrical via of the MEA; (**b**) curing in undesired locations of the photopolymer occurs and the arc of the via is not defined. When the print progresses the incomplete curing of a single layer propagates throughout the entire geometry of the vias resulting in a print failure; (**c**) vias are resolved in the XY-axis with the (**d**) diffraction effect altering the print dimensions in the XY-plane.

Our proposed theory for the observations is provided schematically in Figure 6. At an angle of 90°, the cross-section of the portion being printed (Figure 6a) where the laser prints only an arc of the electrical via of the MEA. As the Z-axis resolution is 25 μm, the sagitta length of the arc would be 8 μm as the radius of the MEA via is 200 μm. The sagitta length of the arc is therefore much smaller than the laser spot size and curing in undesired locations of the photopolymer occurs and subsequently the arc of the via is not defined (Figure 6b). When the print progresses, the incomplete curing of a single layer propagates throughout the entire geometry of the vias resulting in a print failure. This failure is attributed to the fact that the diffraction effects are maximized as the laser light is completely perpendicular to the geometry being printed. In successive layers the diffracted light cures the photopolymer in undesired areas around its spot size and when coupled with the misprinting of the first layer of the vias, the resultant print is a completely closed feature (Figure 5a). This suggests that it would be judicious to print at the lowest angles with respect to the horizontal axis in order for the laser beam to cure the entire via geometry defined in the polymer while it prints each layer. As the vias are resolved in the XY-axis (Figure 6c,d), the diffraction effect will only alter the print dimensions in the XY-plane affecting the design dimensions of the vias and the micro-troughs resulting in print failure of vias less than 400 μm in diameter for a thickness of 1 mm.

In order to experimentally verify our theory, 3D printing of the MEAs were additionally performed at 60°, 45° and 0°. The effect of diffraction leading to false printing is observed to significantly decrease when the angle is lowered to 60° (Figure 5b). Although the electrical vias are open, the micro-troughs for the conducting traces are not well defined due to a reduced feature size (200 μm) as compared to the vias (400 μm). At an angle of 45°, the print quality is significantly improved with all the features being properly defined (Figure 5c). At lower angles (0°), although the device is printed as per the design, we found debris on the surface of the MEA (Figure 5d). The accumulation of the debris which can become a permanent feature on the printed surface can be attributed to the fact that the MEA is now being printed in a completely horizontal direction and the entire MEA surface is making contact with the liquid photopolymer.

We tested our printing theory on a test mironeedle geometry (1000 μm base diameter, 1000 μm height) and printed the features at similar angles (0°, 45°, 60°, 90°) as the MEAs (Figure 5e–h). Interestingly, it is observed that the lagging face of the microneedle geometry is severely misprinted for 90° (Figure 5e) because as one half of the needle is printed, it offers the greatest surface area at the liquid photopolymer interface and the printing would actually be similar to the printing results at 0°. At an angle of 0° with respect to the horizontal, as the microneedle printing starts with the maximum surface area (as opposed to 90° where it starts with the minimum surface) and the entire microneedle surface is damaged thereby giving it the characteristic feature of a ‘burning candle’ as evident from Figure 5h. The surface damages are observed to reduce at 60° (Figure 5f) with a resultant optimum print angle at 45° (Figure 5g). This observation further strengthens our argument of ~45° being the optimum print angle in benchtop SLA 3D printing systems for the 3D designs of biological microdevices.

#### 3.1.2. Effect of Solvent Polishing

**Figure 7:**
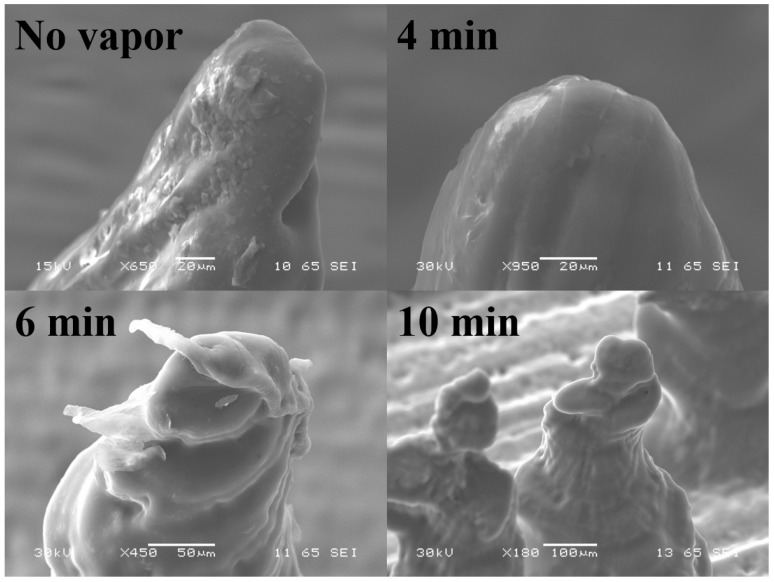
Effect of acetone vapor polishing of the 3D printed microneedles. These MNs were printed at the optimum value of 45°—(**a**) No vapor polishing; (**b**) polishing for 4 min; (**c**) polishing for 6 min and (**d**) polishing for 10 min.

Inherent striations were additionally observed in the microneedles which is a result of the axial resolution being limited to 25 μm. Vapor polishing of the microneedles was performed in order to smoothen the surface of the microneedles as well as to sharpen the microneedle tips. Figure 7 depicts the effect of acetone vapor polishing of the 3D printed microneedles at 45°. The acetone vapor ‘melts’ the photopolymer thereby making the surface of the microneedle smooth while retaining the tip sharpness. However, it was observed that the exposure time to acetone vapor is critical and must be limited (4 min was observed to be the optimum process time for our MN design) to obtain optimum polishing (Figure 7b). Figure 7c,d depict that the MN is severely affected for acetone vapor exposures of 6 min and 10 min respectively.

#### 3.1.3. Effect of Temperature Curing

**Figure 8:**
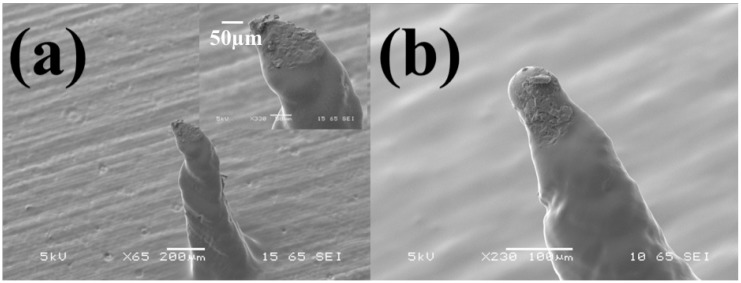
The effect of temperature curing of the microneedles (MNs)—(**a**) No temperature curing and (**b**) curing cycle of 60 °C for 60 min depicting intact tips when subject to a puncturing force of 10 N/patch.

The effect of temperature curing of the microneedles that affects the mechanical properties of the MNs is shown in Figure 8a,b. In the absence of temperature curing the microneedle tip of an optimized geometry is observed to bend with a force of approximately 10 N/patch (Figure 8a). This force translates to approximately 50 MPa per tip which is greater than the Ultimate Tensile Strength (UTS) of human skin [55]. However, after the microneedles are cured (curing cycle of 60 °C for 60 min), their tips remain intact when subject to a similar puncturing force (10 N/patch) (Figure 8b).

### 3.2. MicroElectrode Arrays

**Figure 9:**
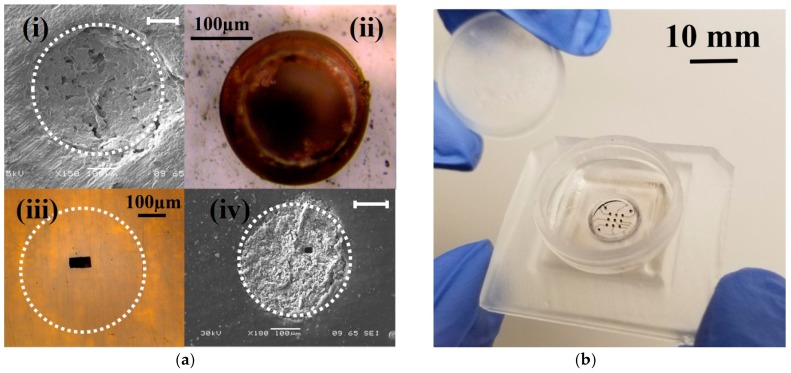
(**a**) SEM and optical micrographs of the (i) non-laminated microelectrode (~400 μm diameter) with the scale bar in figure measuring 100 μm, (ii) the adhesive/PET laminated and micro-drilled electrode (~211 μm diameter), (iii) adhesive/PET laminated and laser micromachined electrode (70 μm × 50 μm in size) and (iv) the SU-8 cast/cross-linked and laser micromachined electrode (30 μm × 30 μm in size) with the scale bar in figure measuring 100 μm respectively. (**b**) Optical microphotograph of a 3D printed MEA after packaging and electroless plating of micro-porous platinum.

**Figure 10:**
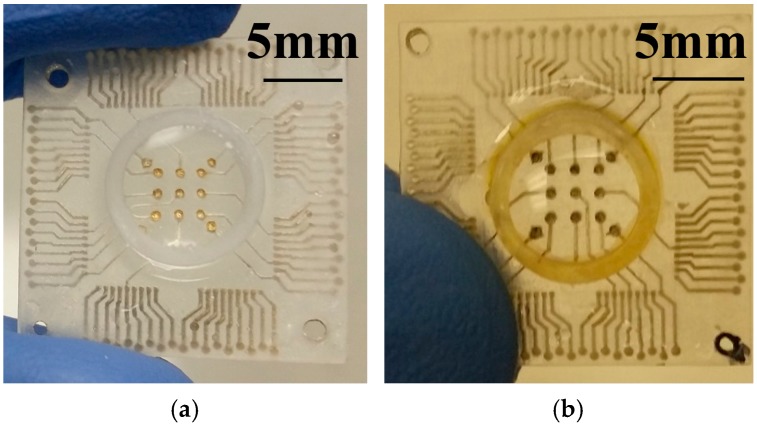
Optical micrographs of the MEA depicting the effect of electroless platinum plating on the ink casted silver electrodes for (**a**) Control sample with no plating and (**b**) 6 h of electroless deposition. A drop of isopropyl alcohol has been intentionally added to enhance the electrode image.

**Figure 11:**
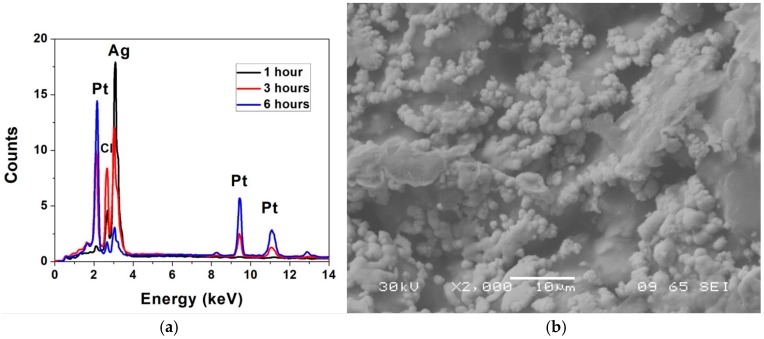
(**a**) EDS measurements confirm the presence of platinum and the coverage on the surface is observed to increase from 7% wt. to 31% wt. and finally to almost 95% wt. after 1 h, 3 h and 6 h of electroless deposition respectively; (**b**) SEM analysis of the electrodes showing micro-porous platinum deposited on the silver paste electrodes after 6 h of electroless deposition.

Figure 9a depicts SEM and optical micrographs of the (i) non-laminated microelectrode (~400 μm diameter); (ii) the adhesive/PET laminated and micro-drilled electrode (~211 μm diameter); (iii) adhesive/PET laminated and laser micromachined electrode (70 μm × 50 μm in size) and (iv) the SU-8 cast/cross-linked and laser micromachined electrode (30 μm × 30 μm in size) respectively. Microelectrodes comparable to standard designs are created with subtractive micromachining. Figure 9b depicts an optical micrograph of a 3D printed MEA after packaging and electroless plating. Figure 10a,b depict the effect of electroless platinum plating on the ink casted silver electrodes. It is clearly seen that the electrodes depict an increased color change from silver to black with an increase in plating time. In order to validate the presence of platinum, SEM and EDS analysis of the electrodes were performed. EDS analysis (Figure 11a) confirms the presence of platinum and the coverage on the surface is seen to increase from 7% wt. to 31% wt. and finally to almost 95% wt. after 1 h, 3 h and 6 h of electroless deposition respectively. Figure 11b shows the SEM image of micro-porous platinum deposited on the electrodes after 6 h of electroless deposition.

**Figure 12:**
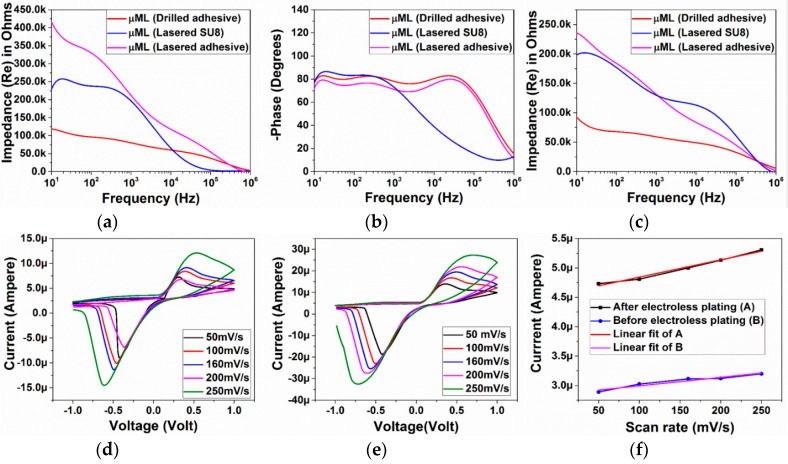
(**a**) Full spectrum impedance and (**b**) phase response of the three distinct types of microelectrodes (average of *n* = 9 of each electrode type) prior to the electroless plating step. (**c**) Average impedance (*n* = 9) of the electrodes after electroless platinum plating for 6 h. Scan rate variation of cyclic voltammetry for the SU-8 cast/crosslinked and laser micromachined electrode (30 μm × 30 μm in size) (**d**) before and (**e**) after electroless plating. (**f**) Current-scan rate plot of the SU-8 cast/crosslinked and laser micromachined electrodes before and after electroless plating with linear fit.

Figure 12a,b depict the full spectrum impedance and phase response for the three distinct types of microelectrodes (average of *n* = 9 of each electrode type) fabricated with the 3D PICLμM process. Interestingly it is observed that the impedance of the adhesive/PET laser micromachined microelectrode (70 μm × 50 μm in size) is greater than that of the laser micromachined SU8 (30 μm × 30 μm in size) electrode prior to the electroless plating step, in spite of it having a higher geometric area. This can be potentially attributed to the incomplete access for the electrolytes to the electrode during the impedance measurement due to the thickness mismatch between the adhesive/PET lamination layer (~63 μm) and the SU8 layer (~5 μm). After the electroless plating step, the effect is however observed to decrease as the micro-porous platinum deposition potentially improves the access of the electrolytes to the electrode area (Figure 12c). This observation is additionally correlated to a similar phase response of the micromilled adhesive/PET lamination and the laser micromachined adhesive/PET lamination.

**Figure 13:**
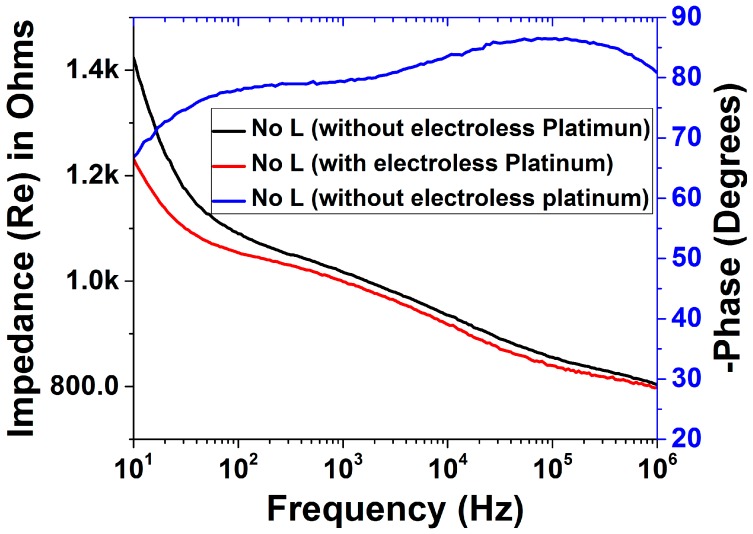
Full spectrum impedance (with and without electroless plating) and phase response (without electroless plating) of the non-laminated microelectrodes that are approximately 400 μm in diameter (average of *n* = 9 of each electrode type).

It is observed that an average impedance of 80 kΩ, 190 kΩ and 194 kΩ is obtained at 1 kHz for the adhesive micro-drilled, adhesive/PET laser micromachined and SU-8 laser micromachined electrodes respectively. Figure 12c shows that the average impedance of the electrodes is reduced to 61 kΩ, 110 kΩ and 140 kΩ at 1 kHz after electroless platinum plating for 6 h. These values are comparable to similar sized microelectrodes fabricated utilizing sophisticated cleanroom-based technologies [14]. Impedance and phase characteristics of the non-laminated microelectrodes are presented in Figure 13. The electrode size in this case is 400 μm as schematically depicted in Figure 1b. These electrodes are ideal for rapid production of relatively large area MEAs.

Cyclic voltammetry of the electrodes has additionally been performed for the smallest microelectrodes (30 μm × 30 μm in size) that we developed using the 3D PICLμM process. To estimate the change in the double-layer interfacial capacitance (C_dl_) of the electrodes after electroless platinum deposition, scan-rate variation of the SU-8 devices were performed (Figure 12d,e). The current/scan rate plot had a linear fit as expected and the capacitance values were extracted from the slope of the graphs (Figure 12f) [14]. It is observed that the capacitance values doubled due to electroless plating from 1.5 μF to 3 μF. This is a significant result demonstrating the potential for our 3D PICLμM microelectrodes in cardiac and neural electrophysiology.

### 3.3. Microneedles

**Figure 14:**
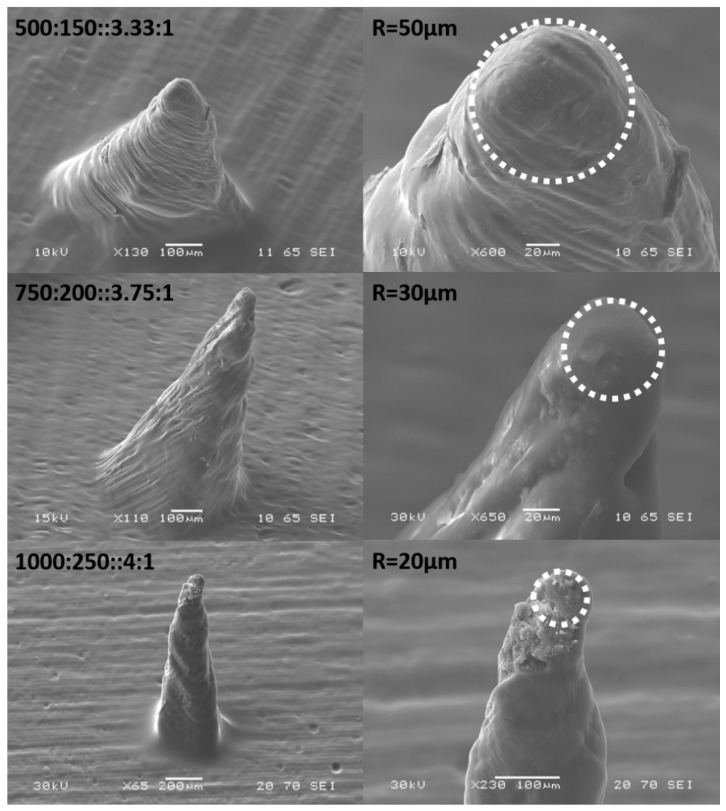
Effect of aspect ratios of 3.33, 3.75 and 4 on the 3D printed needles. Radius of curvature of 50 μm, 30 μm and 20 μm are obtained for aspect ratios of 3.33, 3.75 and 4 respectively.

SEM micrographs in Figure 14 depict the effect of aspect ratio of the 3D printed needles with the dimensions outlined in the Materials and Methods section. It is observed that a radius of curvature of 50 ± 2 μm, 30 ± 3 μm and 20 ± 3 μm are obtained for aspect ratios of 3.33, 3.75 and 4 respectively for an array of 81 microneedles arranged in a 9 × 9 matrix for each of the abovementioned cases.

**Figure 15:**
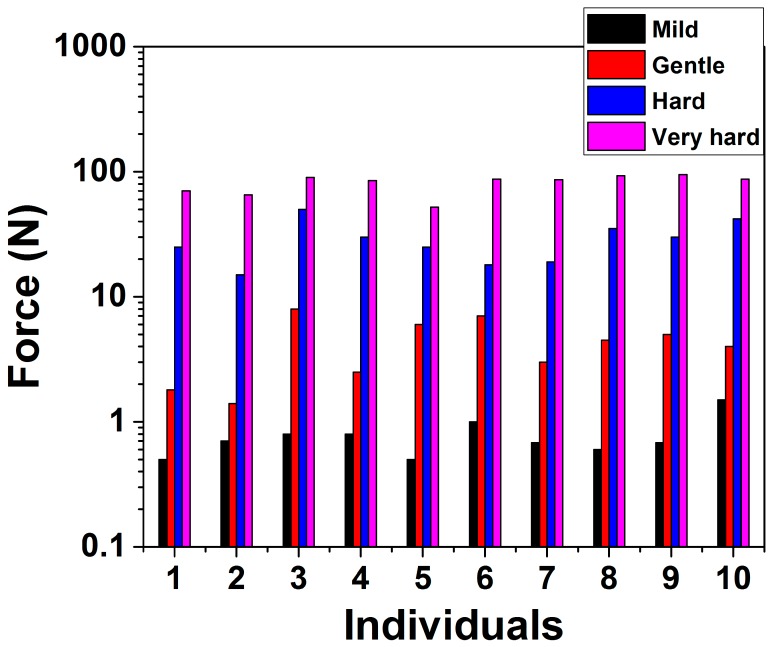
Force application experiments on microneedles (MNs) by 10 volunteers.

The force application experiments performed by 10 volunteers (Figure 15), indicates that a force of approximately 10 N which corresponds to a gentle push onto the skin surface is suitable for transdermal drug delivery by self-administration. This force value compares well with other reports in literature [56].

**Figure 16:**
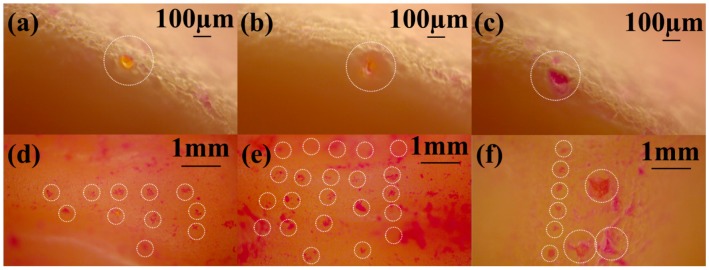
Optical micrographs of MN puncture characteristics on artificial skin—(**a**–**c**): Individual microneedle puncture characteristics at different forces of 0.5 N, 10 N and 30 N respectively; (**d**–**f**) MN array penetration characteristics at different forces of 0.5 N, 10 N and 30 N respectively.

**Figure 17:**
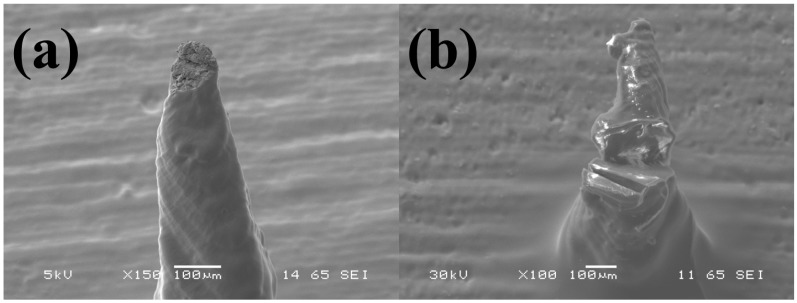
SEM images for the mechanical failure testing of the microneedles (MNs) with (**a**) breakage of the tip observed from forces of ~30 N and a (**b**) complete failure obtained at values of ~40 N.

We further utilized this force value to calculate the pressure on the tip of every microneedle in the array. Values of ~100 MPa, ~50 MPa and ~15 MPa are obtained for radius of curvature of 20 μm, 30 μm and 50 μm respectively for the microneedle patch having 81 individual microneedles. The probability of fracture increases with a smaller needle tip, however larger MN tip may not be sufficiently strong to penetrate the stratum corneum of skin as UTS (Ultimate Tensile Strength) of skin is ~40 MPa [55]. Mechanical analysis and puncturing experiments have therefore been carried out with microneedles having a radius of curvature of 30 μm printed in a 9 × 9 array which we believe are well suited for the end application. Figure 16a–c shows the individual microneedle puncture characteristics at different forces of 0.5 N, 10 N and 30 N respectively. Figure 16d–f show the MN array penetration characteristics. It is clear from this figure that for very low forces (0.5 N) only some of the microneedles penetrate the artificial skin sample as evident with the R6G staining experiments (Figure 16d). Image of an individual puncture site in Figure 16a additionally depicts that the MN was able to barely puncture the skin. For forces in the range of 10 N, the puncture site on the skin has a clear opening corresponding to a dimension of approximately 80 μm (Figure 16b). Moreover, an ordered array with the MN dimensions and pitch is obtained after the staining experiment as depicted in Figure 16e. The distortion in the array is attributed to the curvature of the human skin suture model. For forces in the range of 30 N, the application of the force became non-uniform as the MN patch was being pressed too hard onto the skin surface. This resulted in puncture sites having large openings (Figure 16c) and partial needle array penetration (Figure 16f). Mechanical failure testing was performed with higher values of force to obtain the fracture strength of the microneedles. Breakage of the tip was observed from forces of ~30 N and a complete failure was obtained at values of ~40 N (Figure 17a,b) respectively. The mechanical failure of the microneedles is therefore observed at a significantly higher amount of force (4×) than the force required for its successful operation in transdermal drug delivery.

### 3.4. Microfluidics

**Figure 18:**
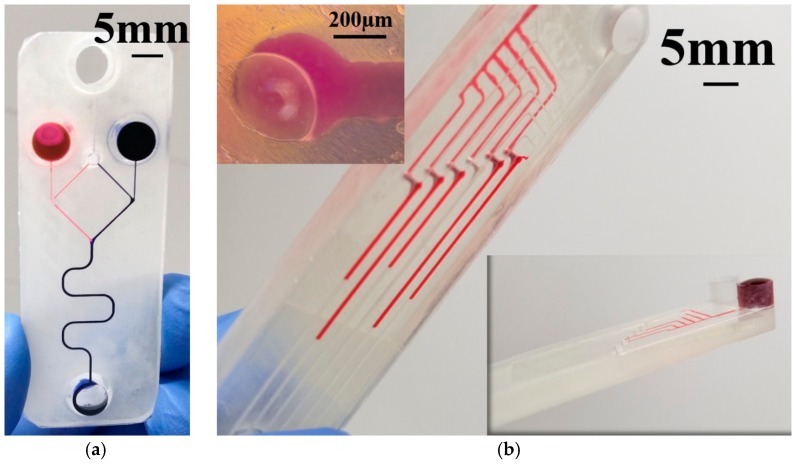
Optical micrographs of the multi-layer microfluidic channels—(**a**) Y-channel with violet (gentian violet) and pink (R6G) colors respectively providing an evidence of micro-mixing; (**b**) functioning of a multi-layer printed microfluidic channel (both top and bottom views are depicted). The liquid making a transition from the top to the bottom microfluidic layers through the microfluidic via of 400 μm is also depicted in the inset.

**Figure 19:**
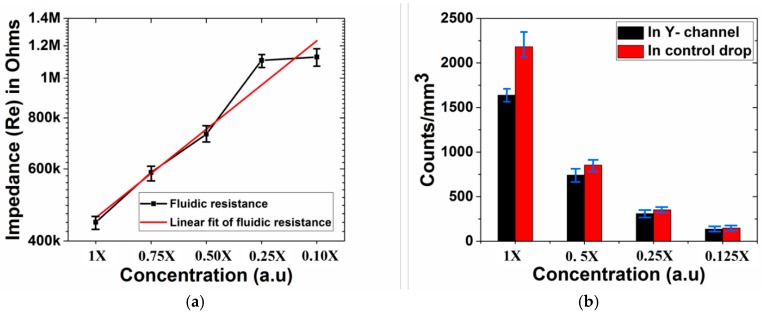
(**a**) Fluidic resistance in the Y-channel for varying concentrations of Dulbecco’s phosphate buffered saline (PBS) buffer with a linear fit of the fluidic resistance for *n* = 3 devices; (**b**) optical counting of the beads for different concentrations of the PS beads in the junction of the Y-channel compared to a polystyrene petri dish (*n* = 3 for both the microfluidic chip and the petri dish control devices).

For the microfluidic devices, the PET/adhesive lamination seals the microfluidic channel from the bottom making it possible to define channel dimensions 150 μm wide and 100 μm deep. It is important to point out here that an opening of 150 μm was successfully defined with the 3D printer as the channel was only 100 μm deep, which corresponds to only 4 printed layers. Other factors such as microscopic contaminants of cured resin as discussed (3D Print Optimization section) additionally limit the accuracy of the final print. Figure 18a,b depict optical micrographs of a monolithically printed microfluidic channel from the top and the bottom after the introduction of flow with Gentian violet and R6G. For the Y-channel (Figure 18a), it is observed that the violet and pink colors respectively of the reagents provides an evidence of mixing, although once the reagents are mixed, the color of the gentian violet begins to dominate the resulting fluid. The functioning of a multi-layer printed microfluidic channel is depicted in Figure 18b. It is observed that the liquid makes an easy transition from the top to the bottom microfluidic layers through the microfluidic via of 400 μm. An optical micrograph of the microfluidic via and the fluid flowing through the via from the top to the bottom layers of the device are additionally shown in the inset. To analyze the behavior of the printed microchannels, fluidic resistance in the Y-channel was obtained by varying the concentration of Dulbecco’s PBS buffer (Figure 19a). A linear fit of the fluidic resistance with concentration is obtained for similar volumes of the injected solution (*n* = 3) in the microfluidic device depicting an ohmic nature as expected [57]. To further study the micromixing capability of the Y-channel in the presence of cell like material (~1.1 μm PS beads), optical counting of the beads was performed for different concentrations of the PS beads in the junction of the Y-channel and compared to a polystyrene petri dish (Figure 19b), which is a gold standard in cell counting and optical imaging assays. It is observed that the measured values (*n* = 3) in the junction of the channel are similar to that of a control drop of beads measured in the petri dish. In fact, for the lower end of concentration (0.125×) the tolerance of the difference is approximately 8%. For the upper end of the concentration (1×), there was observed agglomeration of the PS beads during the flow experiment in the channel which leads to higher values (~35%) of difference with the control and possibilities for counting errors. Nonetheless, a linear decrease in the particle counts is obtained for the varying concentrations of the PS beads in the microchannel with similar results in a PS petri dish demonstrating the ability of our 3D PICLμM devices to perform important microfluidic functionalities in optical assays and cell counting in biological applications.

## 4. Conclusions

Our 3D PICLμM process can result in rapid, robust, benchtop based, design-to-device, cost-effective manufacturing of MicroTAS and BioMEMS devices for low resource settings. We have demonstrated the optimization of the benchtop, SLA 3D printing process that forms the basis of all the realized devices. A print angle of ~45° is optimum for realizing the biological devices described. Solvent vapor polishing and temperature curing are essential for applications where smooth surface finishing and high tensile strength are required. We have demonstrated high yield MEAs having several diameters: 400 μm, 211 μm, (70 μm × 50 μm) and (30 μm × 30 μm) with average 1 kHz impedance of 140 kΩ with a double layer capacitance of 3 μF that are comparable to other demonstrated devices with a micro-porous platinum electrode layer. MNs having a radius of curvature of 30 μm, arranged in a 9 × 9 matrix are demonstrated to obtain an optimum model skin penetration at ~10 N/array with mechanical fracture occurring at ~40 N, depicting high reliability for the intended application. Microfluidic devices having a channel width of 150 μm and depth of 100 μm have also been successfully fabricated and tested for fluidic resistance, micro-mixing, cell-like material imaging and two level fluidic channels connected by microfluidic vias. These devices are produced in the matter of hours from design to a final fabricated device. Our makerspace inspired 3D PICLμM process delivers biological microdevices for a wide variety of applications such as high throughput screening, drug delivery, diagnostics, cell-based assays etc. and is a very attractive alternative to several traditional technologies.

## References

[1] Walsh D.I., Kong D.S., Murthy S.K., Carr P.A. (2017). Enabling microfluidics: From clean rooms to makerspaces. Trends Biotechnol..

[2] Bhattacharjee N., Urrios A., Kang S., Folch A. (2016). The upcoming 3D-printing revolution in microfluidics. Lab Chip.

[3] Farooqui M.F., Karimi M.A., Salama K.N., Shamim A. (2017). 3D-printed disposable wireless sensors with integrated microelectronics for large area environmental monitoring. Adv. Mater. Tech..

[4] Valentine A.D., Busbee T.A., Boley J.W., Raney J.R., Chortos A., Kotikian A., Berrigan J.D., Durstock M.F., Lewis J.A. (2017). Hybrid 3D printing of soft electronics. Adv. Mater..

[5] Vizsnyiczai G., Frangipane G., Maggi C., Saglimbeni F., Bianchi S., Di Leonardo R. (2017). Light controlled 3D micromotors powered by bacteria. Nat. Commun..

[6] Ladd C., So J.H., Muth J., Dickey M.D. (2013). 3D printing of free standing liquid metal microstructures. Adv. Mater..

[7] Gong H., Beauchamp M., Perry S., Woolley A.T., Nordin G.P. (2015). Optical approach to resin formulation for 3D printed microfluidics. RSC Adv..

[8] Gong H., Bickham B.P., Woolley A.T., Nordin G.P. (2017). Custom 3D printer and resin for 18 μm× 20 μm microfluidic flow channels. Lab Chip.

[9] Ota H., Emaminejad S., Gao Y., Zhao A., Wu E., Challa S., Chen K., Fahad H.M., Jha A.K., Kiriya D. (2016). Application of 3D printing for smart objects with embedded electronic sensors and systems. Adv. Mater. Technol..

[10] Nam K.-H., Smith A.S., Lone S., Kwon S., Kim D.-H. (2015). Biomimetic 3D tissue models for advanced high-throughput drug screening. J. Lab Autom..

[11] Konar D., Devarasetty M., Yildiz D.V., Atala A., Murphy S.V. (2016). Lung-on-a-chip technologies for disease modeling and drug development. Biomed. Eng. Comput. Biol..

[12] Rezaei Kolahchi A., Khadem Mohtaram N., Pezeshgi Modarres H., Mohammadi M.H., Geraili A., Jafari P., Akbari M., Sanati-Nezhad A. (2016). Microfluidic-based multi-organ platforms for drug discovery. Micromachines.

[13] Spira M.E., Hai A. (2013). Multi-electrode array technologies for neuroscience and cardiology. Nat. Nanotechnol..

[14] Kim J.-H., Kang G., Nam Y., Choi Y.-K. (2010). Surface-modified microelectrode array with flake nanostructure for neural recording and stimulation. Nanotechnology.

[15] Larraneta E., Lutton R.E., Woolfson A.D., Donnelly R.F. (2016). Microneedle arrays as transdermal and intradermal drug delivery systems: materials science, manufacture and commercial development. Mater. Sci. Eng. R Rep..

[16] Temiz Y., Lovchik R.D., Kaigala G.V., Delamarche E. (2015). Lab-on-a-chip devices: How to close and plug the lab?. Microelectron. Eng..

[17] Wainger B.J., Kiskinis E., Mellin C., Wiskow O., Han S.S., Sandoe J., Perez N.P., Williams L.A., Lee S., Boulting G. (2014). Intrinsic membrane hyperexcitability of amyotrophic lateral sclerosis patient-derived motor neurons. Cell Rep..

[18] Woodard C.M., Campos B.A., Kuo S.-H., Nirenberg M.J., Nestor M.W., Zimmer M., Mosharov E.V., Sulzer D., Zhou H., Paull D. (2014). iPSC-derived dopamine neurons reveal differences between monozygotic twins discordant for parkinson’s disease. Cell Rep..

[19] Qin Y., Howlader M.M., Deen M.J., Haddara Y.M., Selvaganapathy P.R. (2014). Polymer integration for packaging of implantable sensors. Sens. Actuator B Chem..

[20] Kim R., Joo S., Jung H., Hong N., Nam Y. (2014). Recent trends in microelectrode array technology for in vitro neural interface platform. Biomed. Eng. Lett..

[21] Ita K. (2015). Transdermal delivery of drugs with microneedles—potential and challenges. Int. J. Pharm..

[22] Runyan W.R., Bean K.E. (1990). Semiconductor Integrated Circuit Processing Technology.

[23] Chen W., Li H., Shi D., Liu Z., Yuan W. (2016). Microneedles as a delivery system for gene therapy. Front. Pharmacol..

[24] O’Mahony C. (2014). Structural characterization and in vivo reliability evaluation of silicon microneedles. Biomed. Microdevices.

[25] Wang P.M., Cornwell M., Hill J., Prausnitz M.R. (2006). Precise microinjection into skin using hollow microneedles. J. Investig. Dermatol..

[26] Donnelly R.F., Singh T.R.R., Woolfson A.D. (2010). Microneedle-based drug delivery systems: microfabrication, drug delivery, and safety. Drug Deliv..

[27] Indermun S., Luttge R., Choonara Y.E., Kumar P., du Toit L.C., Modi G., Pillay V. (2014). Current advances in the fabrication of microneedles for transdermal delivery. J. Control. Release.

[28] Choi S.-O., Kim Y.C., Park J.-H., Hutcheson J., Gill H.S., Yoon Y.-K., Prausnitz M.R., Allen M.G. (2010). An electrically active microneedle array for electroporation. Biomed. Microdevices.

[29] Aoyagi S., Izumi H., Isono Y., Fukuda M., Ogawa H. (2007). Laser fabrication of high aspect ratio thin holes on biodegradable polymer and its application to a microneedle. Sens. Actuator A Phys..

[30] McAllister D.V., Wang P.M., Davis S.P., Park J.-H., Canatella P.J., Allen M.G., Prausnitz M.R. (2003). Microfabricated needles for transdermal delivery of macromolecules and nanoparticles: fabrication methods and transport studies. Proc. Natl. Acad. Sci. USA.

[31] Park J.-H., Allen M.G., Prausnitz M.R. (2006). Polymer microneedles for controlled-release drug delivery. Pharm. Res..

[32] Han M., Hyun D.-H., Park H.-H., Lee S.S., Kim C.-H., Kim C. (2007). A novel fabrication process for out-of-plane microneedle sheets of biocompatible polymer. J. Micromech. Microeng..

[33] Lippmann J.M., Geiger E.J., Pisano A.P. (2007). Polymer investment molding: method for fabricating hollow, microscale parts. Sens. Actuator A Phys..

[34] Sullivan S.P., Murthy N., Prausnitz M.R. (2008). Minimally invasive protein delivery with rapidly dissolving polymer microneedles. Adv. Mater..

[35] Chu L.Y., Choi S.O., Prausnitz M.R. (2010). Fabrication of dissolving polymer microneedles for controlled drug encapsulation and delivery: bubble and pedestal microneedle designs. J. Pharm. Sci..

[36] Kim Y.-C., Quan F.-S., Compans R.W., Kang S.-M., Prausnitz M.R. (2010). Formulation of microneedles coated with influenza virus-like particle vaccine. Aaps Pharmscitech.

[37] Donnelly R.F., Majithiya R., Singh T.R.R., Morrow D.I., Garland M.J., Demir Y.K., Migalska K., Ryan E., Gillen D., Scott C.J. (2011). Design, optimization and characterisation of polymeric microneedle arrays prepared by a novel laser-based micromoulding technique. Pharm. Res..

[38] Donnelly R.F., McCrudden M.T., Alkilani A.Z., Larrañeta E., McAlister E., Courtenay A.J., Kearney M.-C., Singh T.R.R., McCarthy H.O., Kett V.L. (2014). Hydrogel-forming microneedles prepared from “super swelling” polymers combined with lyophilised wafers for transdermal drug delivery. PLoS ONE.

[39] Huang H., Fu C. (2007). Different fabrication methods of out-of-plane polymer hollow needle arrays and their variations. J. Micromech. Microeng..

[40] Ito Y., Hagiwara E., Saeki A., Sugioka N., Takada K. (2006). Feasibility of microneedles for percutaneous absorption of insulin. Eur. J. Pharm. Sci..

[41] Gittard S.D., Ovsianikov A., Chichkov B.N., Doraiswamy A., Narayan R.J. (2010). Two-photon polymerization of microneedles for transdermal drug delivery. Expert Opin. Drug Deliv..

[42] Nanoscribe GmbH Eggenstein-Leopolds-Hafen, Germany. https://www.nanoscribe.de/en/.

[43] Lu Y., Mantha S.N., Crowder D.C., Chinchilla S., Shah K.N., Yun Y.H., Wicker R.B., Choi J.-W. (2015). Microstereolithography and characterization of poly (propylene fumarate)-based drug-loaded microneedle arrays. Biofabrication.

[44] Lim S.H., Ng J.Y., Kang L. (2017). Three-dimensional printing of a microneedle array on personalized curved surfaces for dual-pronged treatment of trigger finger. Biofabrication.

[45] Glick C.C., Srimongkol M.T., Schwartz A.J., Zhuang W.S., Lin J.C., Warren R.H., Tekell D.R., Satamalee P.A., Lin L. (2016). Rapid assembly of multilayer microfluidic structures via 3D-printed transfer molding and bonding. Microsyst. Nanoeng..

[46] FormLabs Somerville, MA, USA. https://formlabs.com/blog/how-to-post-cure-3d-prints/.

[47] All3DP GmbH München, Bavaria, DE. https://all3dp.com/1/best-resin-dlp-sla-3d-printer-kit-stereolithography/.

[48] Azim N., Sommerhage F., Aubin M., Hickman J., Rajaraman S. (2017). Precision Plating of Electrogenic Cells on Microelectrodes Enhanced with Nano-Porous Platinum for Cell-Based Biosensing Applications.

[49] Karnati C., Aguilar R., Arrowood C., Ross J., Rajaraman S. (2017). Micromachining on and of transparent polymers for patterning electrodes and growing electrically active cells for biosensor applications. Micromachines.

[50] Axion Biosystems Atlanta, GA, USA. https://www.axionbiosystems.com/.

[51] Multi Channel Systems MCS GmbH Reutlingen Germany. http://www.multichannelsystems.com/.

[52] Adafruit Industries LLC New York, NY, USA. https://www.adafruit.com/product/166.

[53] Juodkazis S. (2016). Manufacturing: 3D printed micro-optics. Nat. Photonics.

[54] MatterHackers Lake Forest, CA, USA. https://www.matterhackers.com/news/tech-breakdown-an-in-depth-look-at-the-moonray-s-3d-printer.

[55] Gallagher A., Ní Annaidh A., Bruyère K. (2012). Proceedings of the Dynamic Tensile Properties of Human Skin, IRCOBI Conference 2012, 12–14 September 2012, Dublin (Ireland).

[56] Ripolin A., Quinn J., Larrañeta E., Vicente-Perez E.M., Barry J., Donnelly R.F. (2017). Successful application of large microneedle patches by human volunteers. Int. J. Pharm..

[57] Shipulya N., Konakov S., Krzhizhanovskaya V. (2016). Development and simulation of microfluidic wheatstone bridge for high-precision sensor. J. Phys. Conf. Ser..

